# Chronic Undernutrition Differentially Changes Muscle Fiber Types Organization and Distribution in the EDL Muscle Fascicles

**DOI:** 10.3389/fphys.2020.00777

**Published:** 2020-07-23

**Authors:** Erika E. Rodriguez-Torres, Jorge Viveros-Rogel, Kenia López-García, Enrique Vázquez-Mendoza, Gonzalo Chávez-Fragoso, Salvador Quiroz-González, Ismael Jiménez-Estrada

**Affiliations:** ^1^Center for Research in Mathematics, Hidalgo State Autonomous University (UAEH), Pachuca, Mexico; ^2^Faculty of Health Sciences, Autonomous University of Tlaxcala, Tlaxcala, Mexico; ^3^Department of Physiology, Biophysics and Neuroscience, Center for Research and Advanced Studies, National Polytechnic Institute, Mexico City, Mexico; ^4^Department of Computer Science, Center for Research and Advanced Studies, National Polytechnic Institute, Mexico City, Mexico; ^5^Department of Medical Acupuncture and Rehabilitation, State University of Ecatepec, Ecatepec, Mexico

**Keywords:** fiber type, chronic undernutrition, fractal analysis, distribution functions, machine learning, ATPase, skeletal muscle

## Abstract

Fiber type composition, organization, and distribution are key elements in muscle functioning. These properties can be modified by intrinsic and/or extrinsic factors, such as undernutrition and injuries. Currently, there is no methodology to quantitatively analyze such modifications. On one hand, we propose a fractal approach to determine fiber type organization, using the fractal correlation method in software Fractalyse. On the other hand, we applied the kernel methodology from machine learning to build radial-basis functions for the spatial distribution of fibers (distribution functions), by dividing into square cells a two-dimensional binary image for the spatial distribution of fibers from a muscle fascicle and mounting on each cell a radial-basis function in such a way that the sum of all cell functions creates a smooth version of the fiber histogram on the cell grid. The distribution functions thus created belong in a reproducing kernel Hilbert space which permits us to regard them as vectors and measure distances and angles between them. In the present study, we analyze fiber type organization and distribution in fascicles (F2, F3, F4, and F5) of the *extensor digitorum longus* muscle (EDLm) from control and undernourished male rats. Fibers were classified according to the ATPase activity in slow, intermediate, and fast. Then, (*x, y*) coordinates of fibers were used to build binary images and distribution functions for each fiber type and both conditions. The fractal organization analysis showed that fast and intermediate fibers, from both groups, had a fractal organization within the four fascicles, i.e., the fiber assembly is distributed in clusters. We also show that chronic undernutrition altered the organization of fast fibers in the F3, although it still is considered a fractal organization. Distribution function analysis showed that each fiber type (slow, intermediate, and fast) has a unique distribution within the fascicles, in both conditions. However, chronic undernutrition modified the intra-fascicular fiber type distributions, except in the F2. Altogether, these results showed that the methodology herein proposed allows for analyzing fiber type organization and distribution modifications. On the other side, we show that chronic undernutrition alters not only the fiber type composition but also the organization and distribution, which could affect the muscle functioning, and ultimately, its behavior (e.g., locomotion).

## 1. Introduction

Nature is ordered at all levels, from microscopic (atomic, molecular, and cellular) to macroscopic (individual and population levels), but when a disaster or disease occurs at any level such order changes. The application of mathematics and computer science in anatomical and/or physiological problems has allowed a better and deeper understanding of the fundamental processes of living beings. Cells, tissues, and organs in vertebrates present an organization which is mathematically similar to that observed in other biological systems (e.g., ecosystems) and manifests self-similarity (Mandelbrot, 1983). It is now possible to study the organization of particular biological systems (such as muscles) using fractal tools which have become essential in the work of physicists, chemists, biologists, physiologists, economists, among others. Such tools have allowed researches to reformulate old problems into novel terms, and address complex problems in simplified forms (Liebovitch et al., 1987; Jelinek and Fernandez, 1998; Reese et al., 2012; Hernández and Menéndez-Conde, 2013).

The skeletal muscle is a heterogeneous tissue composed of various fiber types, which can be classified according to their metabolic and contractile characteristics as glycolytic and oxidative or slow, intermediate, and fast fibers, respectively (Ariano et al., 1973). The organization of muscle fibers is relevant to maintain the homeostasis and muscle functioning. This organization can be altered by disease, inadequate nutrition, exercise or injury, modifying their contractile and structural properties. Yet we found practically no studies oriented to investigate the organization of fiber types in skeletal muscles and how natural or pathological conditions can modify it, specifically in the case of the *extensor digitorum longus* muscle (EDLm), which is composed in four fascicles (F2, F3, F4, and F5) with different fiber composition, metabolism, and size (Balice-Gordon and Thompson, 1988; Kissane et al., 2016; Vázquez-Mendoza et al., 2017). This particular muscle participates in the extension of toes (each fascicle extends a single toe, the F2 extends toe 2, the F3 extends toe 3, and so on) and in the dorsiflexion of the ankle in the rat.

Recently, it has been illustrated elsewhere that chronic undernutrition exerts a differential effect on the relative fiber type composition in the EDLm fascicles (Vázquez-Mendoza et al., 2017). Particularly, it was observed that the third fascicle (F3) was more affected than the others, being the sequential order of effects as follows: F3>F5>F4=F2. In that study, the authors suggested that those changes in the relative composition of fiber types in the EDLm fascicles could induce modifications in the intra-fascicle fibers organization. One way to analyze this is by mathematical methods such as fractal estimation analysis, which can determine whether a fiber phenotype group is organized in clusters or spread randomly over the whole muscle or fascicle.

Besides the organization, fiber types distribution within a muscle is crucial to its functioning (Burkholder et al., 1994). The visualization method that we developed consists in the application of the kernel methodology from machine learning to build distribution functions for the spatial localization of fiber types. In brief, on the reconstructed microphotograph of the stained section, we superimposed a square grid of size *N* and built a histogram for each fiber type. For each cell, we built a Gaussian kernel function that is mainly supported therein, by taking into account the number and localization of fiber types within the cell. The individual cell functions are then linearly superimposed to obtain a function whose graph resembles a smoothing of the histogram of the fiber type under study, we call it the distribution function (DF) of the fiber type shown in the histological image. DFs obtained in this way belong in a (finite-dimensional) reproducing kernel Hilbert space, which in effect enables us to treat each one of them (and thus each image) as vectors and thus measure distance and angle between any pair of them. We then use distance and angle measurements (which we call dissimilarity quantifiers) to differentiate data images from one another and in turn quantify the effects of undernutrition in EDLm fiber content.

In this study, we aimed to develop a methodology to evaluate changes in fiber type organization and spatial distribution due to alterations provoked by traumatic processes such as spinal cord injury, motor nerve damage, multiparity, or by metabolic diseases (undernutrition or obesity), among others. To this end, we applied fractal estimation analysis to analyze fiber type organization and we were able to make a quantitative analysis of changes in the structure of muscle fibers due to chronic undernutrition. On the other hand, we applied the kernel methodology from machine learning to build distribution functions for the spatial localization of fiber types and along with the dissimilarity quantifiers we were able to assess how chronic undernutrition alter the fiber type distribution within the EDLm fascicles.

## 2. Materials and Methods

All experiments were performed in accordance with the Guide for the Care and Use of Laboratory Animals (National Research Council, 2010; National Institutes of Health, Bethesda, MD, USA; Animal Welfare Assurance #A5036-01). The animal protocols were approved by the Institutional Bioethical Committee for the Care and Handling of Laboratory Animals (UPEAL-Protocol 013−02, CINVESTAV).

### 2.1. Animals

Chronic undernutrition protocol. We used nulliparous female Wistar rats (257.4 ± 16.3 g body weight), which were randomly allocated in two groups: control (C, *n* = 16) and undernourished (U, *n* = 18). Control group had free access to commercial food (Formulab 5008; LabDiet, Framingham, MA, USA); while the undernourished group was fed with 50% of the mean food intake given to control animals, both groups had access to water *ad libitum*. Two weeks after, female rats of both groups (C and U) were put together with a male for 1 week in the same cage to ensure mating and the consecutive pregnancy. After that, males were removed. The day females gave birth litters were adjusted to nine pups: five males and four females. During gestation, birth, and lactation all rats remained on the same feeding protocol to which they were subjected from the beginning (C or U). Each mother and her offspring were housed in large acrylic cages (43 × 53 × 20 cm). After weaning (postnatal day 21), pups remained on the same feeding protocol as their mothers (C or U) until the experimental proceeding. Later, male rats were housed individually in acrylic cages (32 × 47 × 20 cm) under the same conditions of light/dark cycle (12/12 h) and temperature (22–24°C). No supplementary mineral, trace elements or vitamins were added to the food supply of undernourished animals. Further details of these protocols can be found in Ruiz-Rosado et al. (2013) and Vázquez-Mendoza et al. (2017).

At 35 postnatal days, we randomly selected 6 males coming from each experimental group (C, *n* = 6; and U, *n* = 6), which weighed and anesthetized with urethane (1.6*g*/*kg* of body weight). The EDL muscles were quickly removed and weighed (more details Vázquez-Mendoza et al., 2017). Subsequently, the four fascicles of each muscle (*F*2 to *F*5) were carefully separated and their length measured. After that, fascicles were immersed in 2−methylbutane, cooled to near freezing point with liquid nitrogen and stored at −80°C until their processing. At the end of tissue extraction, the animals were euthanized using an overdose of anesthetic (urethane). Subsequently, the middle segment of each fascicle was sectioned and mounted on a specimen holder in a cryoprotectant solution (Tissue−TekⓇ O.C.T Compound, SakuraⓇ Finetek, Torrance, Ca). Serial transverse sections (10 μ*m* thick) of each specimen tissue were obtained by means of a cryostat at −25°C (CM−1520; Leica Biosystems, Nussloch, Germany). The sections were subsequently mounted on glass coverslips for staining.

### 2.2. Histoenzymatic Analysis

EDLm fascicle sections were stained with the myofibrillar alkaline ATPase activity technique (pH = 9.4, modified from Guth and Samaha, 1970) to identify the fast, intermediate and slow fiber types. In brief, the muscle sections were submerged 20 min in a pre-incubation solution (0.01 M Tris base and 0.018 M *CaCl*_2_, pH 10.3), then they were washed three times for 5 min with deionized water and subsequently incubated at 37°C for 60 min in the incubation solution (1.5% w/v of adenosine-5′-triphosphate in pre-incubation solution, pH 9.4). After incubation, slides were washed for 3 min with 0.2 M *CaCl*_2_ and transferred to 2% w/v *CoCl*_2_ solution for another 5 min. Subsequently, they were washed ten times with deionized water and finally transferred to 10% v/v ammonium sulfide for 3 min. Stained sections were washed, dehydrated with ascending alcohol solutions and mounted with glycerogel and coverslips. Photomicrographs of each muscle fascicle were taken by a digital camera (AxioCam MRc, Zeiss, Germany) mounted on a microscope (Olympus CX31, NY, US). The whole muscle fascicle was reconstructed with the photomicrographs using Photoshop CS4. After that, the spatial position (*x, y* coordinates) of each and the total number of the different fiber types was determined in control and undernourished muscles using ImageJ (Rasband, 2011). According to the alkaline ATPase technique, the fibers were identified as light = slow, Type I; gray = fast, Type IIb and dark = intermediate, Type IIa/IId (see section 7).

### 2.3. Experiments With Synthetic Data

In order to determine if the dissimilarity quantifiers (distance and angle) can differentiate between highly similar distribution, we generated synthetic data which we considered to be challenging to the quantifiers. Below is describe in detail how these distribution were constructed. This was implemented in a MatLab script available in https://github.com/GonzaloCin/DistributionFunctions.

#### 2.3.1. Data Generation

Our synthetic data consists of four collections of randomly generated points and with very pronounced tendencies toward spreading and/or clustering. Each set has a geometric shape which we think should be challenging for the algorithm to discern one set from the another. The shapes are: a ball (uniform spread in all directions with no tendency toward clustering), a ring (uniform spread in all directions, with a pronounced tendency to cluster far from and uniformly around the global centroid of the set), a set in the shape of a sum sign (with pronounced spreading and clustering tendencies along the coordinate axes), a cross-shaped data set (same as the sum-shaped set but with a 45-degree rotation angle).

To generate each data set we first generated *Q* points (*x, y*) on a square [−*L*/2, *L*/2] × [−*L*/2, *L*/2], using a uniform distribution, then points were selected according to the following criteria:

*Ball:* only points satisfying *x*^2^ + *y*^2^ ≤ *L*^2^/4 were chosen to form part of the data set.

*Ring:* only points satisfying (*L*^2^/2 − δ)^2^ ≤ *x*^2^ + *y*^2^ ≤ *L*^2^/4 were chosen.

*Sum sign:* only points satisfying −δ/2 ≤ *x* ≤ δ/2 or −δ/2 ≤ *y* ≤ δ/2.

*Cross:* only points (ξ, η) satisfying −δ/2 ≤ ξ ≤ δ/2 or −δ/2 ≤ η ≤ δ/2 were chosen and then rotated a 45-degree angle: $x=\left(\xi -\eta \right)/\sqrt{2}$ and $y=\left(\xi +\eta \right)/\sqrt{2}$.

**Figure 1:**
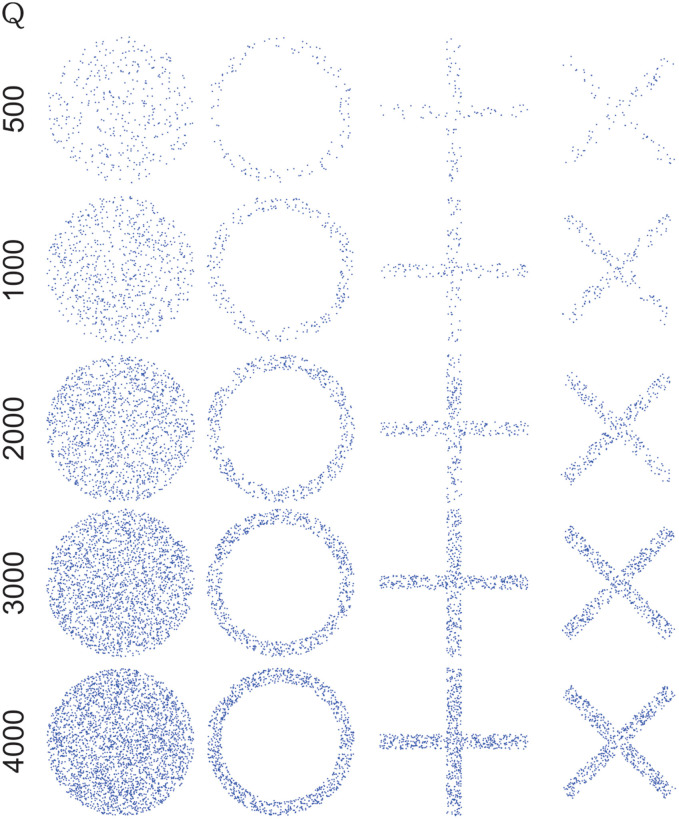
Artificial data generated choosing δ=, *L*=, and *Q* = 500, 1,000, 2,000, 3,000, 4,000. For each value of *Q* a collection of four data sets was generated (one for every shape: ball, ring, sum, cross) and for every one of them a distribution function was built. More details of experiments with synthetic data in section 2.3. Note that by increasing the number of points (*Q*), the shape of figures is more defined and, in dissimilarity quantifiers, induce an increase of distance, although angle is not affected, see text in section 7.2.

We chose δ = *L*/9, and *Q*= 500, 1,000, 2,000, 3,000, 4,000 (Figure 1). For each value *Q* a collection of four data sets was then generated (ball, ring, sum, cross) and for every one of them a distribution function was built using the methodology in 4. For every pair of distributions in each collection, distances and angles were calculated using formulae (*S*19) and (*S*20) from Supplementary Material, respectively. The results are reported in 7.2.

## 3. Fractal Correlation Integral Method

To determine the fractal structure of fiber types in fascicles we used the Fractalyse software (Thomas et al., 2008) and binary images constructed. This freeware program has been developed by Frankhauser and colleagues and can be downloaded on the website http://www.fractalyse.org/. The original version of this software has been developed in the frame of the French research program “Ville émergente,” financed by the PUCA (Plan Urbanisme Construction Architecture). Correlation analysis turned out to be the most reliable method as it introduces fewer artifacts compared to others, such as grid and dilation analysis. In fractal “correlation analysis,” each fiber type pixel is surrounded by a small square window of size ε. The number of fibers pixels within each window is then counted. This allows the mean number of pair correlations per window *N*(ε) to be computed. This step is repeated for windows of increasing size. It results in a series of points that can be represented on a Cartesian graph where the *X*−*axis* refers to the size of the window ε = (2*i*+1) (*i* being the iteration step), and the *Y*−*axis* refers to the mean number of points per window. The next step consists in fitting this empirical curve to a theoretical curve that corresponds to a fractal law, i.e., a power law that links the number of correlations *N*(ε) to the size of the window ε:

(1) $$\begin{array}{r} N=\varepsilon^{D} \end{array}$$

The exponent *D* is the fractal dimension, or in this case, the correlation dimension. However, real-world patterns cannot strictly follow a fractal law. Therefore, it is useful to introduce a generalized fractal law, which contains two additional parameters:

(2) $$\begin{array}{r} N=\text{a}\varepsilon^{D}+c \end{array}$$

The parameter *a* is called the “pre-form factor.” It is giving a synthetic indication of local deviations from the estimated fractal law (Frankhauser, 1993, 1998; Thomas et al., 2007). For a mathematical fractal structure, is to be equal to 1 (Gouyet, 1996). Experience shows that when it goes 4 or less than 0.1, a fractal pattern is not confirmed (see Thomas et al., 2007). In real-world patterns, fractal behavior may change across scales. Changes often occur within rather small values of ε, i.e., for small distances, often corresponding to the clusters of fibers of the same type. In order to avoid local effects and hence wrong estimations, it is useful to introduce an additional parameter *c* that allows the correct estimation of *D* and *a* (Frankhauser, 1998; Thomas et al., 2008). The software Fractalyze was used to estimate the parameters mentioned above; it is mainly dedicated in this paper to the fractal analysis of fibers types and scaled in such a way that the pixel size is really the counting unit for ε. This ensures that the numbers N(ε) are correctly counted in spatial structures like that illustrated further in this paper. Sensitivity analyses were performed to explore the role and the physiological meaning of *a* when estimating fractal dimensions for EDLm fibers under different conditions. The results were compared with a simplified version of the generalized law (2), where *a* is forced to one:

(3) $$\begin{array}{r} N=\varepsilon^{D}+c \end{array}$$

*D* is often estimated by using a double logarithmic representation of the power law. Nonlinear regression was used to estimate the parameters that best fit the empirical curve since this avoids implicit assumptions about local deviations from the fractal law. Noise is assumed to be an independent additional effect. The fractal dimension *D* of fibers types can take any value between 0 and 2. When *D* = 2, the pattern of a fiber fiber type of EDLm is uniform, following a one-scale logic (Euclidian forms); *D* = 0 corresponds to a pattern made up of a single point (e.g., one or few muscle fibers); and finally, when *D* is between 1 and 2, the elements distributed in clusters over the space. Fractal dimension can be considered as a measure of an object's ability to fill the space in which it resides.

The quality of the estimation is measured by computing the ratio:

(4) $$\begin{array}{r} \frac{cov\left(N^{\left(est\right)},N^{\left(obs\right)}\right)}{\sqrt{var\left(N^{\left(est\right)}\right)var(N^{(obs)}}} \end{array}$$

Where *N*^(*est*^^)^ corresponds to the set of estimated values and N^(*obs*)^ to the observed values. We here call this ratio *R*^2*^ by analogy with the determination coefficient. For values close to 1, N^(*est*)^(ε) and N^(*obs*)^(ε) curves tend to be equal, which means that the fractal model fits well to the observed data. If the fit between the two curves (empirical and estimated) is poor, we can conclude either that the pattern is not fractal or that it is multifractal (e.g., Tannier and Pumain, 2005). In our case, all analyzed patterns lead to *R*^2*^ values >0.99.

## 4. Construction of Distribution Functions

### 4.1. Overview

The work discussed in this section is motivated by the old problem of extracting information about an underlying phenomenon from a collection of direct or indirect measurements or observations of the phenomenon itself, in order to estimate a functional dependency. Concrete examples of this type of problem are, determining which gene is responsible for a certain disease (microarray data classification; Cristianini and Shawe-Taylor, 2000; Schölkopf et al., 2004), and face and handwriting recognition (pattern classification; Duda et al., 2012; Devroye et al., 2013). The theory developed around this type of problems is nowadays known as Learning Theory (Vapnik, 1998; Cucker and Zhou, 2007) and its application was boosted thanks to the accessibility of modern computers capable of performing fast calculations.

Put plainly, the exact problem we are concerned here with is that of fitting a spatial distribution function to a finite set of points on the plane. The reader can think that the coordinates (*x, y*) of those points on the plane, give the location of muscle fibers in the histological image of a transverse muscle section. For distribution function, we will understand a smoothing of the histogram on the plane for the centered data, built on a square grid of given size *N*. *N* is a parameter specified by the user. If Φ:ℝ^2^ → ℝ is the distribution function of a set of points, Φ(***z***) gives the approximate count of points per unit square length at the location ***z*** = (*x, y*) on the plane. Our distribution functions will be linear superpositions of Gaussian kernel functions, one kernel function per each square of the grid. This section we will elaborate on it.

Gaussian kernel functions are a type of radial basis functions (RBFs). Aside from their ample use in classification problems in Bioinformatics, RBFs are also used in a variety of scenarios, such as approximation and interpolation problems (cf. Buhmann, 2003), or in the construction of Lyapunov functions for the determination of the stability of fixed points of certain dynamical systems (cf. Giesl, 2007). The RBF construction method we apply uses the so-called “kernel trick,” which consists in taking advantage of properties of kernel functions to deal with the computational problem that entails high-dimensional data (which is not the case of our data), and to guarantee that the distribution functions so built will belong in an inner-product space which we denote by $L_{N}$, and is the precursor of a Reproducing-Kernel Hilbert Space (the latter being the completion of the former under the norm induced by the inner product (cf. Schölkopf et al., 2004; Wendland, 2005). Working within an inner-product space will allow us to treat functions as vectors and thus measure distance and angle between two functions, we will then use these measurements to make a quantitative assessment of how distinct distribution functions associated to two fiber types are, which will directly translate into a semi-quantitative assessment of how two fiber types with distinct metabolic and myosin ATPase activities distribute across a muscle section.

Below we limit ourselves to presenting the methodology by which distribution functions are built for a single collection of finitely many points. The set of points is thought of as representing the spatial localization of fiber centroids in a given histological image of a muscle fascicle. In order to measure distance and angle between two functions (and thus between two collections of points or images), it is necessary to construct all distribution functions to be compared, simultaneously. The latter can be done by slightly tweaking the construction we present first.

In the next subsection, we briefly describe the construction of distribution functions. For a detail explanation on the mathematical framework of the construction and in what sense it is possible to speak of distance and angle between two distribution functions (see Supplementary Material).

**Figure 2:**
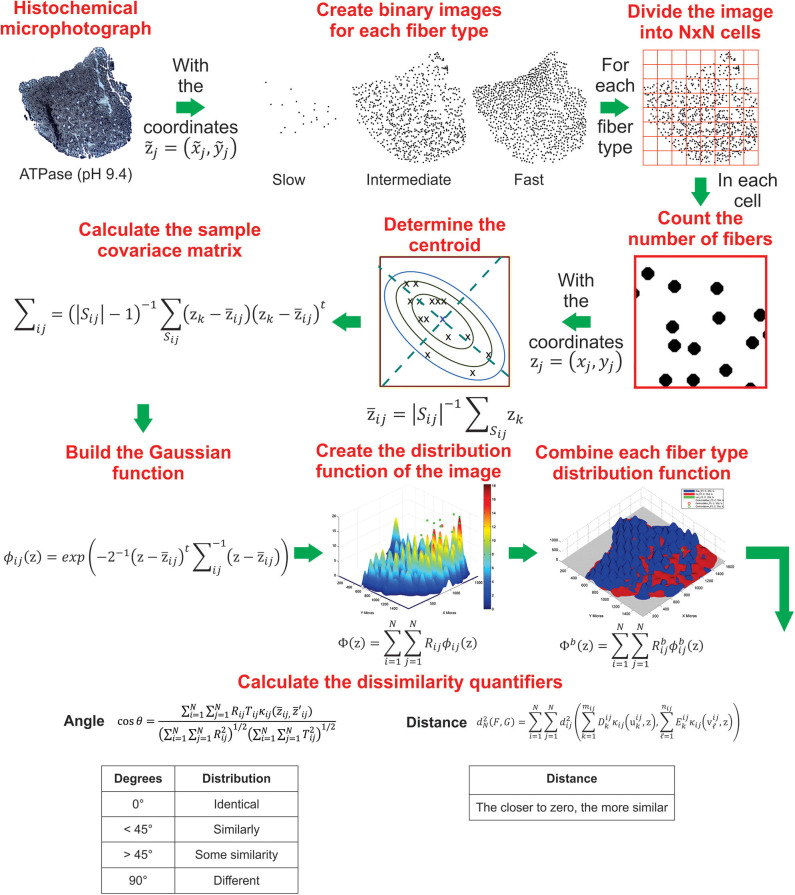
Schematic representation of the sequential steps used to create the muscle fiber type distribution and calculate dissimilarity quantifiers by using the distribution method implemented in MatLab. More details of the construction of distribution functions are indicated in section 4.

Figure 2 shows a schematic representation of how distribution functions are constructed from the (*x, y*) coordinates of muscle fiber type. This method has already been implemented in a MatLab script for the construction of distribution function for a single and for a batch of images, which can be downloaded from https://github.com/GonzaloCin/DistributionFunctions.

### 4.2. Distribution Function for a Single Image

Let $\left\{{\tilde{z}}_{1},\ldots ,{\tilde{z}}_{\nu }\right\}\subset {\mathbb{R}}^{2}$, ${\tilde{z}}_{j}=\left({\tilde{x}}_{j},{ỹ}_{j}\right)$, be a collection of finitely many coordinate pairs of points within a sample image, each one of which represents a muscle fiber of the same type as all other fibers in the collection. Let $\bar{z}=\nu^{-1}\overset{\nu }{\underset{j=1}{\sum }}{\tilde{z}}_{j}$ be the centroid of the collection and *S* = {***z***_1_, …, ***z***_ν_} with $z_{j}={\tilde{z}}_{j}-\bar{z}=\left(x_{j},y_{j}\right)$ be the centered collection of coordinate pairs. *S* is our data set and it is contained within a compact box B=[a,A]×[b,B], where *a* = −ε+min*x*_*j*_ and *A* = ε+max*x*_*j*_, with ε a small, positive, chosen number (*b* and *B* defined similarly). B may as well be determined by the dimensions of the sample image, thus skipping the “cropping” just described.

Choose a fixed positive integer *N* and consider uniform partitions *P* = {*a*_0_, …, *a*_*N*_} and *Q* = {*b*_0_, …, *b*_*N*_} of [*a, A*] and [*b, B*], respectively. So *a*_*k*_ = *a*+*k*(*A*−*a*)/*N* (*b*_*k*_ defined similarly). *P* and *Q* define a uniform rectangular grid over B, composed of *N*^2^ cells, *C*_*ij*_ = [*a*_*i*−1_, *a*_*i*_] × [*b*_*j*−1_, *b*_*j*_]. We next describe a method to mount a bivariate Gaussian function on each cell *C*_*ij*_. Such functions are then superimposed linearly so that the graph of the combined function will look like a smoothed version of the histogram defined on the grid.

Consider a cell *C*_*ij*_ and let *S*_*ij*_ be the subcollection of data points within it, *S*_*ij*_ = {***z***_*k*_∈*C*_*ij*_}⊆*S*. Let |*S*_*ij*_| be the cardinality of *S*_*ij*_ and ${\bar{z}}_{ij}=|S_{ij}{|}^{-1}\underset{S_{ij}}{\sum }z_{k}$ be the centroid of the points in *S*_*ij*_. The sample covariance matrix of points in *C*_*ij*_ is defined as (cf. Duda et al., 2012, p. 90),

(5) $$\begin{array}{r} \Sigma_{ij}={\left(|S_{ij}|-1\right)}^{-1}\underset{S_{ij}}{\sum }\left(z_{k}-{\bar{z}}_{ij}\right){\left(z_{k}-{\bar{z}}_{ij}\right)}^{t}. \end{array}$$

Assume for the moment Σ_*ij*_ is invertible (the case when Σ_*ij*_ is singular is discussed at the end of this section). The bivariate Gaussian function referred to above, also called *Gaussian kernel function* (see next section), is defined as

(6) $$\begin{array}{r} \phi_{ij}\left(z\right)=\exp-2^{-1}{\left(z-{\bar{z}}_{ij}\right)}^{t}\Sigma_{ij}^{-1}\left(z-{\bar{z}}_{ij}\right), \end{array}$$

We now associate to the collection *S* the following *distribution function,* where *R*_*ij*_ = |*S*_*ij*_|,

(7) $$\begin{array}{r} \Phi \left(z\right)=\overset{N}{\underset{i=1}{\sum }}\overset{N}{\underset{j=1}{\sum }}R_{ij}\phi_{ij}\left(z\right). \end{array}$$

Φ is the main object of this section. We now mention three situations that must be sorted out when constructing Φ.

- If *C*_*ij*_ is empty, we set ϕ_*ij*_ = 0 (indentically zero function).
- If *C*_*ij*_ contains only one point ***z***_*p*_ = (*x*_*p*_, *y*_*p*_), we compute its distance δ = max{*a*_*j*_−*x*_*p*_, *b*_*j*_−*x*_*p*_} to the boundary of *C*_*ij*_, redefine $\Sigma_{ij}={\left(\delta /3\right)}^{2}Id_{2}$ where *Id*_2_ is the 2 × 2 identity matrix, and construct ϕ_*ij*_ as in Equation (6), that is $\phi_{ij}\left(z\right)=\exp\left(-9\cdot 2^{-1}\delta^{-2}||z-z_{p}|{|}^{2}\right)$ (||·|| denotes Euclidean distance). The unlikely case in which *C*_*ij*_ contains only one point which lies exactly on its boundary, can be treated in several ways. One way is to associate that point to the adjacent cell of shared boundary, assuming such cell has at least one other point. Another way is to arbitrarily set δ equal to a very small predefined positive number, so that the contribution of ϕ_*ij*_ to the sum Φ is highly localized for its double contribution to the sum in Equation (7) to be significant. We never incurred in this scenario in our experiments.
- If Σ_*ij*_ is singular or nearly so (its determinant is smaller than a pre-established number ε_0_ > 0), we apply the following four steps:
  - let $\lambda_{1}=\underset{S_{ij}}{\max}||z_{k}-{\bar{z}}_{ij}|{|}^{2}$, and λ_2_ = λ_1_/9.
  - pick any ***z***_*k*_ in *C*_*ij*_ and define $u_{1}=\left(z_{k}-{\bar{z}}_{ij}\right)||z_{k}-{\bar{z}}_{ij}|{|}^{-1}$, then if *u*_1_ = (*v, w*) let *u*_2_ = (−*w, v*) so that $u_{1}^{t}u_{2}=0$.
  - Let *M* = [*u*_1_*u*_2_] be a 2 × 2 matrix with columns *u*_1_ and *u*_2_, in that order. and Λ = diag(λ_1_, λ_2_) be a diagonal matrix.
  - Redefine $\Sigma_{ij}=M\Lambda M^{t}$ and construct ϕ_*ij*_ as in (6).

In the next subsection, we establish that the function Φ in (7) belongs in an inner-product space.

For more details on the method of construction (see Supplementary Material).

### 4.3. Distribution Functions for a Batch of Images

In the previous subsection, we described a methodology to fit one distribution function to one single image. In this section, we show that with a slight modification the methodology can be applied to fit a distribution function for every image in a finite collection of *r* distinct images, in tandem, for cross comparison. The meaning of having a set of *r* images depends on the context. For instance, if the points ***z***_*k*_ represent the location of muscle fibers, every image could represent muscle fibers of one of *r* different types. Once a distribution function has been obtained for every image, we wish to calculate distance and angle between pairs of them and assess if those measurements reflect the classification independently established.

The trick now is to make sure that the distribution functions of the images we want to compare, *belong in the same inner-product space* $L_{N}$. In order to achieve that we only need to slightly change our definition of the functions ϕ_*ij*_ in (6). More precisely, we need to modify the definition of the covariance matrix Σ_*ij*_ in (5) as we now describe.

Similarly, as in the previous section, we divide every image into *N* × *N* cells. Let $C_{ij}^{b}$ denote cell (*i, j*) of image *b* and $z_{k}^{b}$ the *k*th point in that image. Let $S_{ij}^{b}=\left\{z_{k}^{b}\in C_{ij}^{b}\right\}$ (set of points of image *b* in its cell $C_{ij}^{b}$), and let $\left|S_{ij}^{b}\right|$ represent the number of points in $C_{ij}^{b}$. We define the *global sample covariance matrix for cell* (*i, j*) as follows:

(8) $$\begin{array}{r} \Sigma_{ij}={\left(S_{ij}-1\right)}^{-1}\overset{r}{\underset{b=1}{\sum }}\underset{S_{ij}^{b}}{\sum }\left(z_{k}^{b}-{\bar{z}}_{ij}\right){\left(z_{k}^{b}-{\bar{z}}_{ij}\right)}^{t}, \end{array}$$

where $S_{ij}=\overset{r}{\underset{b=1}{\sum }}|S_{ij}^{b}|$ and ${\bar{z}}_{ij}$ is the *global centroid for cell* (*i, j*),

(9) $$\begin{array}{r} {\bar{z}}_{ij}=S_{ij}^{-1}\overset{r}{\underset{b=1}{\sum }}\underset{S_{ij}^{b}}{\sum }z_{k}^{b}. \end{array}$$

Thus, if

(10) $$\begin{array}{r} \phi_{ij}^{b}\left(z\right)=\exp\left(2^{-1}{\left(z-{\bar{z}}_{ij}^{b}\right)}^{t}\Sigma_{ij}^{-1}\left(z-{\bar{z}}_{ij}^{b}\right)\right), \end{array}$$

then

(11) $$\begin{array}{r} \Phi^{b}\left(z\right)=\overset{N}{\underset{i=1}{\sum }}\overset{N}{\underset{j=1}{\sum }}R_{ij}^{b}\phi_{ij}^{b}\left(z\right) \end{array}$$

is the distribution function for sample image *b*, with $R_{ij}^{b}=|S_{ij}^{b}|$. The same observation as in equation (11) may be applied in this case for an alternate choice for the coefficients *R*_*ij*_. We can also adapt cases (*i*), (*ii*), and (*iii*) in the first section to deal with the scenarios in which Σ^−1^ is singular or nearly so.

## 5. Data Analysis

In this work, we used the data obtained in a previous study from our group (Vázquez-Mendoza et al., 2017) corresponding to slow, intermediate, and fast fibers in the EDLm fascicles of control and undernourished young rats (35 days old).

### 5.1. Fiber Type Fractal Organization

In order to assess the fractal organization of the fiber types in the fascicles of the EDLm, we constructed binary images using the (*x, y*) coordinates of each fiber type with a MatLab program developed in our laboratory. These images were then analyzed using the fractal correlation method in Fractalyse, obtaining the fractal dimension (*D*), pre-form factor (*a*), and parameter *c*. From this analysis, we excluded the slow fibers because of their small number in muscle sections.

### 5.2. Fiber Type Distribution Functions

Intra-fascicle distribution of fiber types was determined applying the method described in the previous section (4), implemented in a MatLab script. In brief, this method requires the (*x, y*) coordinates of each fiber type, that are used to create a binary image, which is divided into *N* × *N* cells. In our case, we used an *N* = 11 because in a pilot study we observed that this number of cells allowed us to have cells with a few, many and a large number of fibers. This is relevant due to it lets us visualize the distribution of fibers in an optimal resolution, showing how the fibers form groups and how these groups are distributed within the muscle. Then in each cell, the number of fibers is counted in order to calculate the estimator covariance matrix and the centroid. Subsequently, a Gaussian function is built for each cell. Next, the distribution function of the fiber type is created by the lineal superposition of all Gaussian functions. Finally, the distribution function of each fiber type are merged to visualize them in a single image (Figure 2). Also, the (*x, y*) coordinates are used to calculate the dissimilarity quantifiers, distance (*D*), and angle (θ), which allows to compare two distribution functions. On one hand, the closer the distance to zero, the more similar are the distributions. On the other hand, angles <45° indicates similar distributions, whereas angles >45° indicates dissimilar distributions (Figure 2).

In order to determine differences between experimental condition (control and undernourished), we calculated the dissimilarity quantifiers for each pair of distributions within a fascicle (i.e., slow vs. fast, slow vs. intermediate, and intermediate vs. fast), which we called intra-fascicular fiber type distributions. Also, we calculated the dissimilarity quantifiers for synthetic data comparing between shapes (ring vs. ball, ring vs. cross, ring vs. sum, ball vs. cross, ball vs. sum, and cross vs. sum) and all quantities of points (500, 1,000, 2,000, 3,000, and 4,000 points).

## 6. Statistical Analysis

Dissimilarity quantifiers of fiber types distribution, as well as the fractal dimension (*D*), *a*, and *c* indexes between control and undernourished EDLm fascicles, were analyzed performing unpaired Student's *t*-test or Mann–Whitney test, depending on its normality, evaluated with the Kolmogorov–Smirnov test. Dissimilarity quantifiers of synthetic data comparisons were analyzed using Person's correlation. Data analysis was performed in GraphPad Prism (v.6.00, GraphPad Sofware, Ca., USA). Significant differences were considered at *P* ≤ 0.05. Data are showed as mean ± S.E.M.

## 7. Results

The data obtained in this study was partially reported in Vázquez-Mendoza et al. (2017), where we found that chronic undernutrition reduces the percentage of intermediate fibers in the F4, increases this fiber type in the F5 and reduces the fast fibers in the F3 and F5, compared to control fascicles (Table 1). According to the changes in the proportion of fiber types it could be established the following sequence of fascicles affected by chronic undernutrition (most to less): *F*3 > *F*5 > *F*4 = *F*2. According to the later, Vázquez-Mendoza et al. (2017) proposed that chronic undernutrition evokes a differential effect on the relative proportion of fiber types in EDLm fascicles and suggested that such condition may provoke changes in the intra-fascicle distribution and organization of fiber types.

**Table 1:** Percentage fiber composition and fractal organization parameters [*a* index, fractal dimension (*D*), and *c* index] corresponding to the intermediate and fast fiber types present in the different EDLm fascicles (F2, F3, F4, and F5) of control (C) and undernourished (U) rats.

	**Composition (%)**					
	**Intermediate**				**Fast**	
	**C**	**U**			**C**	**U**
F2	57.6 ± 4.5	57.6 ± 5.2			31.7 ± 5.4	37.9 ± 5.4
F3	51.9 ± 5.4	67.2 ± 4.0			46.0 ± 5.5	29.7 ± 4.1*
F4	63.6 ± 2.3	53.6 ± 3.6*			33.3 ± 2.5	42.2 ± 4.0
F5	45.2 ± 2.0	54.4 ± 1.1*			53.7 ± 2.0	44.9 ± 1.1*
	**a** **index**					
	**C**	**U**			**C**	**U**
F2	0.18 ± 0.03	0.22 ± 0.01			0.15 ± 0.03	0.21 ± 0.04
F3	0.20 ± 0.03	0.26 ± 0.03			0.22 ± 0.03	0.43 ± 0.11
F4	0.35 ± 0.10	0.26 ± 0.04			0.27 ± 0.09	0.25 ± 0.07
F5	0.16 ± 0.01	0.24 ± 0.07**			0.25 ± 0.07	0.28 ± 0.02
	**Fractal dimension (D)**					
	**C**	**U**			**C**	**U**
F2	1.77 ± 0.05	1.80 ± 0.01			1.75 ± 0.03	1.70 ± 0.05
F3	1.78 ± 0.02	1.75 ± 0.03			1.73 ± 0.03	1.50 ± 0.08*
F4	1.75 ± 0.04	1.78 ± 0.02			1.68 ± 0.04	1.74 ± 0.02
F5	1.84 ± 0.01	1.81 ± 0.01			1.80 ± 0.01	1.81 ± 0.01
	**c** **index**					
	**C**	**U**			**C**	**U**
F2	6.54 ± 1.00	5.08 ± 1.01			7.48 ± 1.39	6.72 ± 0.54
F3	4.51 ± 2.18	0.30 ± 3.01			5.50 ± 1.22	6.62 ± 2.48
F4	−2.71 ± 4.87	2.02 ± 2.75			7.66 ± 3.07	4.98 ± 2.08
F5	6.65 ± 0.90	0.11 ± 1.42**			−1.81 ± 1.66	0.75 ± 1.01

Mean ± S.E.M. Significant differences between C and U were determined using Student's t-test or Mann–Whitney test. *P < 0.05, **P < 0.01. n = 6, except in Intermediate F2C, F2U, F4U, and in Fast F3U, where n = 5, and in Fast F2 C, n = 4.

### 7.1. Fractal Analysis Results

In order to perform the fractal analysis, we first estimated the *a* index, which needs to be >0.1 and <4.0 to treat them as fractals. Fast and intermediate fibers in all fascicles (control and undernourished) showed an *a* in the range of a fractal (Table 1; only five values were excluded from the analysis because they showed *a* < 0.1). Although intermediate fibers in the fascicle F5 of the undernourished group showed a significant larger *a* value than the control one (*P* < 0.01) these fibers are still in range. With these results, we were able to treat the individual binary images of fast and intermediate fibers from the control and undernourished rats as fractals. In the case of slow fibers, these were excluded from the analysis due to the lower number of fibers.

Once established that the binary images can be treated as fractal, we estimated the dimension parameter (*D*), which gives us how the fibers are organized within the fascicles. The results showed that fast and intermediate fiber in all EDLm fascicles, of both conditions, had similar *D* values, varying between 1.5 and 1.84 (Table 1), indicating that both intermediate and fast fibers are distributed in clusters over the transverse area of each fascicle. Statistical analysis showed that chronic undernutrition only reduced the fractal dimension of fast fibers in the F3, as compared to control (C, 1.73 ± 0.03 vs. U, 1.50 ± 0.08; *P* < 0.05; Table 1). Nonetheless, this value still represents a distribution in clusters over the transverse area of the fascicle.

In real-world patterns, fractal behavior may change across scales. Changes often occur within rather small values of ε, i.e., for small distances, often corresponding to the clusters of fibers of the same type. In order to avoid local effects and hence wrong estimations, it is useful to introduce an additional *c* parameter that allows the correct estimation of *D* and *a* values (Table 1). Similar to the *a* index, the *c* parameter of fast and intermediate fibers in all fascicles was similar between the groups (*P* > 0.05; Table 1), except for the intermediate fibers in the undernourished F5, which showed a significant reduced *c* value as compared to control (C, 6.65 ± 0.90 vs. U, 0.11 ± 1.42; *P* < 0.01) that could be related with the reduction of the muscle CSA due to undernutrition.

Altogether, this analysis showed that intermediate and fast fibers in the four EDLm fascicles (F2, F3, F4, and F5) of the control rats present a fractal organization within the fascicle, i.e., the fiber assembly is distributed in clusters. Also, it showed that this organization is preserved during chronic undernutrition, except for the fast fibers in the F3, whose organization is reduced, but still conserve the distribution in clusters. This probably helps to preserve the optimal muscle functioning despite the alimentary condition.

### 7.2. Distribution Functions Results

Although the fractal organization was preserved in all fascicles of undernourished rats, the fiber types distributions could be modified, in order to determine changes in the fiber types distribution we constructed the distribution function of slow, intermediate, and fast fibers of control and undernourished rats.

Before analyzing the distribution function of fiber type, we aimed to determine if our dissimilarity quantifiers could differentiate between very similar distributions. To achieved this, we calculated the dissimilarity quantifiers for synthetic data (Figure 1) that present distributions that could be a challenge to our quantifiers. Tables 2, 3 contain comparison results with synthetic data, angle measurements are displayed in degrees. In general, we observed an increase in distance values while increasing the number of points (*R*^2^ = 0.99, *P* < 0.001, in all comparisons, except Ring vs. Ball, where *R*^2^ = 0.67, *P* > 0.05), which did not happen with the angle values (Ring vs. Ball, *R*^2^ = 0.72; Ring vs. Cross, *R*^2^ = 0.20; Ring vs. Sum, *R*^2^ = 0.37; Ball vs. Cross, *R*^2^=0.75; Ball vs. Sum, *R*^2^=0.75; Cross vs. Sum, *R*^2^=0.48; *P* > 0.05, in all cases), of the shapes being compared. Our discussion of these results is given in section 8.2.

**Table 2:** Distance (*D*) and angle (θ) for artificial data.

**Synthetic data**	**Ring vs. Ball**		**Ring vs. Cross**		**Ring vs. Sum**	
***Points***	**D**	**θ**	**D**	**θ**	**D**	**θ**
500	44.44	75.59	27.51	83.19	29.32	84.46
1, 000	78.36	65.58	57.34	85.50	58.10	80.50
2, 000	144.88	64.10	101.98	85.42	107.54	82.27
3, 000	203.90	59.17	151.85	83.38	155.11	79.78
4, 000	273.52	60.36	193.62	82.81	210.09	80.88
*Mean*	149.02	64.96	106.460	84.06	112.03	81.58
*S*.*E*.*M*.	92.82	6.50	67.68	1.30	72.87	1.85

*500, 1,000, 2,000, 3,000, and 4,000 points. Ring vs. ball, ring vs. cross, and ring vs. sum*.

**Table 3:** Distance (*D*) and angle (θ) for artificial data.

**Synthetic data**	**Ball vs. Cross**		**Ball vs. Sum**		**Cross vs. Sum**	
**Points**	**D**	**θ**	**D**	**θ**	**D**	**θ**
500	42.91	73.19	44.89	77.61	27.77	83.37
1, 000	81.59	70.73	84.05	72.62	56.61	82.33
2, 000	145.53	65.04	145.22	64.35	99.69	83.08
3, 000	214.80	64.81	209.16	61.80	135.39	79.66
4, 000	285.31	65.06	283.28	63.85	192.16	82.63
*Mean*	154.03	67.77	153.32	68.05	102.32	83.01
*S*.*E*.*M*	98.23	3.93	95.69	6.76	64.84	2.78

*500, 1,000, 2,000, 3,000 and 4,000 points. Ball vs. cross, ball vs. sum, cross vs. sum*.

#### 7.2.1. Intra-fascicle Distribution of Slow, Intermediate, and Fast Fiber Types

**Figure 3:**
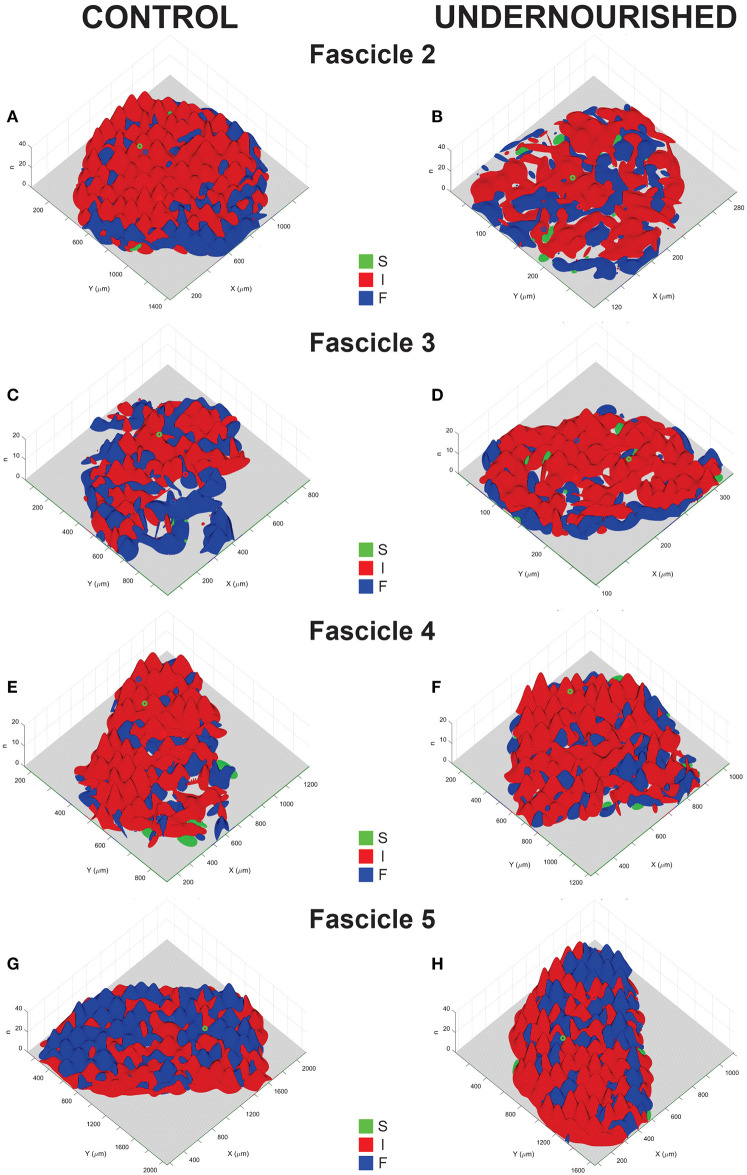
Representative distribution functions maps of slow (S), intermediate (I) and fast (F) fibers in the EDLm fascicles (F2, F3, F4, and F5) from control **(A, C, E, G)** and undernourished **(B, D, F, H)** rats. The *X-axis* *Y*−*axis* represent the length and width of the cross-section of the fascicles, whereas the *Z*−*axis* represents the number of fibers (n). Qualitatively, we can observe that each fascicle present a characteristic distribution pattern of each fiber type, which is modified by chronic undernutrition, more evident in the F3. Quantitatively, dissimilarity quantifiers showed that, indeed, intra-fascicular distributions in F3, F4, and F5 are altered, becoming both, more similar and more dissimilar, for details see text.

To visualize the intra-fascicle distribution of fibers types, binary density histograms from sections of EDLm fascicles of control and undernourished rats stained with the alkaline ATPase technique were constructed (Figure 3). Apparently, most of the undernourished EDLm fascicles (F2, F4, and F5) showed a similar distribution of fiber types as that of control fascicles (Figures 3A,B,E–H). In contrast, the internal distribution of fiber types in the F3 of undernourished rats (Figure 3D) completely differs from that determined in the control group (Figure 3C).

Once established that the dissimilarity quantifiers are robust to differentiate between distributions, we compared the fiber types distribution between fascicles of control and undernourished male rats, in order to obtain not just a qualitative comparison. Considering that our fascicles sections did not have anatomical orientation and in order to avoid misinterpretation of the results, we compared the intra-fascicular distribution of fiber types and then we compared this values between groups (Table 4).

**Table 4:** Distance (*D*) and angle (θ) of the intra-fascicular fiber type distributions (slow vs. intermediate, *SvsI*; slow vs. fast, *SvsF*; intermediate vs. fast, *IvsF*) in the EDLm fascicles (F2, F3, F4, and F5) of control (C) and undernourished (U) rats.

		**SvsI**		**SvsF**		**IvsF**	
		**D**	**θ**	**D**	**θ**	**D**	**θ**
**F2**	**C**	59.20 ± 5.39	68.39 ± 0.72	38.95 ± 3.49	72.84 ± 0.93	57.58 ± 3.75	63.98 ± 1.32
	**U**	53.97 ± 1.97	71.46 ± 1.17	35.47 ± 1.32	75.38 ± 0.56	48.32 ± 137	55.05 ± 1.45
**F3**	**C**	82.20 ± 6.50	72.76 ± 1.39	68.09 ± 2.71	75.88 ± 0.68	71.75 ± 3.45	50.15 ± 1.16
	**U**	60.00 ± 3.42	75.98 ± 1.23	27.12 ± 0.84 ***	80.71 ± 0.91	56.89 ± 3.28	65.53 ± 0.96**
**F4**	**C**	105.01 ± 3.86	80.81 ± 0.74	55.72 ± 2.19	79.62 ± 0.32	116.64 ± 3.91	58.41 ± 1.25
	**U**	66.45 ± 4.03*	76.31 ± 1.80	53.57 ± 4.53	77.99 ± 1.09	49.60 ± 2.09 *	46.02 ± 1.61
**F5**	**C**	105.76 ± 4.87	74.07 ± 0.76	122.37 ± 2.31	78.67 ± 0.37	83.23 ± 2.65	40.27 ± 0.71
	**U**	167.50 ± 3.10**	81.24 ± 0.26**	143.62 ± 2.61 *	81.71 ± 0.46	95.07 ± 2.78	34.13 ± 0.80

Mean ± S.E.M. Significant differences between C and U were determined using Student's t-test or Mann–Whitney test. *P < 0.05, **P < 0.01, ***P < 0.001. n = 6, except in F4U, where n = 5.

F2 intra-fascicular distributions from the control animals showed distances lower than 60, and angles greater than 45 degrees, suggesting that each fiber type has a distinctive distribution within the fascicle (Table 4). And this was similar to the undernourished animals, suggesting that chronic undernutrition does not affect the fiber types distribution of the F2.

In the case of F3, in the control group, we found that distance was between 60 and 90, whereas angles were greater than 45 degrees, indicating that, as with F2, each fiber type distribution is dissimilar to the others (Table 4). In contrast, in the undernourished group showed distances smaller than the controls, particularly in the distribution between slow and fast fibers (*P* < 0.001; Table 4). In the case of angles, they were also greater than 45 degrees, but the angle between the distribution of intermediate and fast fiber was greater than the control (*P* < 0.01). These results suggest that chronic undernutrition modified the fiber type distribution in the F3, but maintaining differences among them.

For the F4 in the control group, distances were between 50 and 120, but all angles were greater than 45 degrees, suggesting that the fiber types distribution are dissimilar among them (Table 4). On the other hand, chronic undernutrition reduced the distances between the slow and intermediate fiber (*P* < 0.05; Table 4) and between the fast and intermediate fiber (*P* < 0.05; Table 4). Likewise, chronic undernutrition decreased the angles, especially between the fast and intermediate fibers (*P* < 0.05; Table 4). This suggests that chronic undernutrition affects the fiber type distribution of the F4, making them more similar among them.

Finally, the F5 of control animal showed distances between 80 and 130, but the angles were grater than 45 degrees between the distribution of slow and intermediate fibers and between slow and fast fibers (Table 4). In contrast, the angles between intermediate and fast fibers distribution were lower than 45 degrees, indicating that fast and intermediate fibers distribution is more similar between them than with the slow fiber distribution. Contrary to F4, in F5, chronic undernutrition increased the distance, especially between the slow and intermediate fibers distributions (*P* < 0.01) and between the slow and fast fibers distribution (*P* < 0.05; Table 4). Also, increased the angle values, notably between the slow and intermediate fibers distributions (*P* < 0.01; Table 4). This indicates that chronic undernutrition, also modifies the intra-fascicular fibers distribution in the F5, making the slow fiber distribution more dissimilar to the intermediate and fast fibers distributions.

Altogether, these results suggest that chronic undernutrition has a differential effect not just in the fiber type composition, but also in the organization and distribution of the fiber types. And these changes could affect the muscle function and ultimately the behavior (e.g., locomotion).

## 8. Discussion

Here, we have developed a methodology to compare fiber types organization and distribution in the EDLm fascicles of control and undernourished rats. On one hand, we determined that intermediate and fast fibers in the EDLm fascicles present a fractal organization, i.e., they are distributed in clusters over the transverse area of each fascicle. Likewise, our results showed that chronic undernutrition reduces significantly the fractal organization of fast fibers in the F3, but preserving the organization in clusters. On the other hand, the distribution functions showed that each fiber phenotype has a unique spatial distribution pattern, but chronic undernutrition modifies the intra-fascicular fiber types distributions in the F3, F4, and F5.

### 8.1. Distribution Function Method

Before discussing the biological data, we make a detailed analysis regarding the methods developed by other authors in previous work on the spatial distribution, especially those based on the calculation of Dirichlet tessellations and adjacency matrices (cf. Venema, 1991, 1995; Grotmol et al., 2002) and correlation dimension (cf. Arsos and Dimitriu, 1995).

Methods based on Dirichlet tessellations (and variations of them) as well as adjacency matrices are particularly useful when one wants to distinguish between two or more classes of points, one of which is scarce in comparison to the others. Dirichlet tessellations provide a strictly *visual* tool when one wishes to establish clustering among two or more classes of points within one single image, as well as to *show* a tendency or pattern in the spread of those classes. However, this method is subjective in the sense that it is the user who decides whether or not there is clustering or spread, and in what direction. In other words, there is no established criterion or numerical parameter by which several users may all agree on the presence or absence of clustering or a pattern within the data sets. Moreover, such methods are mainly restricted to two-dimensional data.

On the other hand, calculation of the correlation dimension is a *quantitative* task which is not constrained to points on the plane. The concept of correlation dimension was introduced in Grassberger and Procaccia (1983a) within the context of dissipative dynamical systems whose phase space evolution is driven by the presence of a strange attractor. This type of attractors arise when the flow of the system is contracting in some directions, expanding in others, and confined within a compact region. This causes the volume element to fold on itself and acquire a multi-sheeted shape with a Cantor-like (self-similar) structure in certain directions, which is directly reflected on the attractor. The latter structures are typically associated to fractal sets (cf. Grassberger and Procaccia, 1983b).

To calculate the correlation dimension, one starts with a sequence of points on the attractor, {***z***_*j*_:*j* = 1, …, *M*}, which ideally is a time series with fixed time increment time τ, i.e., ***z***_*j*_ = ***z***(*t*+*jτ*). A measure of the spatial correlation of the points is the quantity (cf. Grassberger, 1983; Grassberger and Procaccia, 1983b):

(12) $$\begin{array}{r} C\left(\ell \right)=\underset{M\to \infty }{\lim}\frac{1}{M^{2}}\times \left\{\text{number of pairs}\left(j,k\right)\text{such that}||z_{j}-z_{k}||<\ell \right\}. \end{array}$$

For small values of ℓ, it was established in Grassberger and Procaccia (1983b) that

(13) $$\begin{array}{r} C\left(\ell \right)\sim \ell^{\nu }, \end{array}$$

where the constant ν is the correlation dimension or correlation exponent (cf. Grassberger, 1983; Grassberger and Procaccia, 1983b). The correlation dimension ν is related to Kolmogorov's capacity *D*, also called box-counting dimension or even “fractal dimension” after Mandelbrot (cf. Mandelbrot, 1977), but the latter terminology is misleading as there is more than one way to define the dimension of a fractal set (cf. Grassberger, 1983). In Grassberger and Procaccia (1983b), *D* is identified as the Hausdorff dimension, but the latter has a more involved definition (cf. Farmer et al., 1983). Mandelbrot used the term fractal dimension in reference to the Hausdorff dimension and to the information dimension σ, as follows: ν ≤ σ ≤ *D*. ν and *D* solely depend on the metric of the phase space for their calculation, whereas σ also requires defining a probability measure. ν should not exceed, for instance, the embedding dimension of the attractor (i.e., if the ***z***_*j*_'s are *p*-dimensional vectors, then ν should not be greater than *p*).

In applications (e.g., measurements obtained from observations carried out in a laboratory), however, only a finite set of measurements will be available and thus considering the limit in Equation (12) is not possible. In that case Equation (12) is replaced by the following:

(14) $$\begin{array}{r} C\left(\ell \right)=\frac{2}{M\left(M-1\right)}\times \left\{\text{number of pairs}\left(j,k\right)\text{such that}||z_{j}-z_{k}||<\ell \right\}; \end{array}$$

observe that the coefficient 2/*M*(*M* − 1) is the reciprocal of the maximum number of different pairs of data points. When using Equation (14) *M* is *expected* to be (sufficiently) large and the relation Equation (13) is still *assumed* to hold approximately for ℓ ∈ *I*, where *I* is an open interval contained in (δ_*min*_, δ_*max*_) with δ_*min*_ the minimum distance between two data points and δ_*max*_ the maximum distance (the diameter of the point set). In the context of this work, which is the same as that of Arsos and Dimitriu (1995), these two hypotheses are important. Eckmann and Ruelle (1992) shows how to estimate the number of points necessary in order for Equation (14) to yield a meaningful result. Our method of construction of distribution functions does not need those hypotheses.

We now discuss the meaning of the exponent ν in Equation (13). Recall that Equation (13) is a law that is assumed to hold approximately for the right-hand side of (Equation 14), for ℓ values within a certain interval *I*. Even though the following two cases will not be meaningful to us, they are nonetheless convenient to discuss for the sake of clarity. If there is only one pair of points at a distance δ from each other, that is *M* = 2, then *C*(ℓ) = 0 for ℓ ≤ δ and *C*(ℓ) = 1 if ℓ > δ. In such case one can consider that ν = 0 as ℓ^0^ = 1. When there is only one point in the data set, ν is *defined* as zero. Now, in general, when the data set is finite, but large (*M* large), it is customary to estimate ν by plotting log*C*(ℓ) against logℓ and adjusting a line over the range of values of ℓ for which a linear tendency is observed, such tendency is expected to be easily detected for *M* sufficiently large. This, thus can be considered the criterion by which to consider that the size of the data set is adequate for purposes of the study. In this scenario, ν gives the growth rate of the number of data pairs at a distance no greater than ℓ. When ν = 1, the growth is considered “neutral,” that is, no clear tendency toward either spreading or clustering among data points can be declared. When 0 < ν < 1, this means a slower-than-linear growth in the number of pairs of points at a distance at most ℓ, in other words, one can speak of a tendency in the data toward clustering. On the contrary, when ν > 1, it means an accelerated growth (greater than linear) in the number of points at a distance at most ℓ that is, the data points are spreading (no clustering). We think this interpretation is the most honest conclusion one can derive from the calculation of the correlation dimension alone, in the context of this work (and that of Arsos and Dimitriu, 1995), that is, outside of the dynamical systems context.

Pullen (1977a,b) uses a simple quantitative technique to analyze fiber composition and distribution of the adult tibialis anterior muscle in rats. The author considers complete cross-sections of the muscle in different specimens and sets deep-superficial and medial-lateral axes. Histological images are then projected on a counting grid. Only those cells of the grid along the axes are considered. The magnification of each image is set so that fifty to one hundred fibers are shown in each cell and their identification is possible. For each cell along the axes the fiber ratio (fiber type over total number of fibers in the cell) is determined for three different types of fibers which the authors name IIA, I, and IIB, and correspond to intermediate, slow and fast fibers, respectively, according to their oxidative, phosphorylase, and ATPase histochemistry. Then, for each cell, histograms are constructed to appreciate fiber distribution and muscle composition. The variance among histograms is then analyzed. The results obtained showed that, not only distribution of fiber histochemical types varies across an entire cross-section of the muscle, but also the histochemical technique employed seems to affect the quantitative analysis. Fiber cross-section area is also calculated, in an attempt to verify disparities found among classification of fibers based on different histochemical techniques. The author concludes with a few important observations such as fiber classification based on different histochemical techniques may produce different distributions profiles.

The relevance of Pullen (1977a,b) to our study comes from the fact that it considers a division of the cross-section of a muscle, by the deep-superficial and medial-lateral axes. Along these axes, cells of a counting grid are considered. Within each cell of this grid, a relative count of fiber types is performed and a profile of each fiber type distribution is revealed, by drawing histograms put together with the counts of all cells along each axis. The distribution profiles obtained by the author sweep along the perpendicular directions of the axes, not across the entire muscle. Our study is more focalized as it considers fascicles, but it does so entirely. We also construct distribution functions which we later use to be able to compare among fiber types and try to establish parameters for their distinction (distance and angle between pairs of them).

In another study, Henriksson-Larsén et al. (1983) focused on the importance of defining the biopsy depth when analyzing the distribution of different types of fibers of human skeletal muscle (tibialis anterior). Two types of fibers were considered based on enzyme histochemical classification criteria: type 1 (red, slow-twitch, and oxidative) and type 2 (white, fast-twitch, and glycolytic). The authors report significant variations in the relative number of fibers depending on the depth of the muscle biopsy (human tibialis anterior). There are two main reasons why Henriksson-Larsén et al. (1983) seems relevant to our study:

1. It brings about the question of whether biopsy depth should be a factor to be taken into consideration. Given the size of the specimens used in our work, this point does not seem relevant.
2. In Figure 2, the authors have a histogram of fibers type 2, followed (Figure 2D) by a contour plot. For these figures, a grid was drawn on every mounted section of a muscle. Each cell of the grid drawn on the image had a side length of 1mm and was divided into nine sub-cells. The total number of fibers for each type was determined for the central sub-cell only (one-ninth of the whole cell). However, the authors do not detail how they determine the size of the grid or the method used to obtain the contour map (for the latter they used a computer program, but they did not mention which one). The authors use these contour plots to visually assess the distribution of fibers on a cross-section of the muscle, but they do not try to establish a method to compare two distributions (as we did in our work), nor do they attempt to give a more formal definition to the term distribution (as we did in our work).

In Wang and Kernell (2001), advantage is taken of the match between motoneuronal nerve endings and their muscle fibers, so that studying the spatial distribution of the latter will translate into properties of the spatial distribution of the former. To that end, the authors devise two methods for determining the position and the extent of muscle fibers within a muscle cross-section: the “mass vector method” and the “sector method.” The authors developed these methods in order to get on the subject of degree and direction of what they call fiber type regionalization, something that had been missed by previous studies which are more focused on providing a detailed or pointwise description of the muscle fiber distributions (Johnson et al., 1973; Pullen, 1977a,b; Armstrong and Phelps, 1984). The specific questions that the methods developed by the authors address are: how much does the center for a given fiber type population differ from that for the muscle as a whole? (vector method) and, how much of the available cross-section space of a muscle is utilized by a given fiber type? (sector method). The vector method designed by the authors allows two things. On one hand, it allows pointing at a specific region within the muscle being observed, in which certain type of fibers are distributed. This is done by constructing the “mass vector,” which is a vector that points from the centroid of the muscle section to the centroid of the fiber set. On the other hand, by scaling the mass vector by the diameter of a circle, whose area is that of the cross-section of the muscle, one obtains the “fiber target vector.” The latter can be compared in length and in magnitude with other fiber target vectors from similar samples. Both, the mass and the fiber target vectors, account for the general location of the set of fibers as a whole, within the muscle. To account for the extent of the fiber set within the muscle cross-section, the authors designed the sector method. As in the case of the convex hull method, the section method determines what percentage of the total cross-section area of the muscle is being covered by the fibers under study. Unlike the convex hull method, the sector method tends to exclude regions of the cross-section that are not populated by fibers, and which would otherwise be included in the convex hull method due to the irregularity of the fiber set perimeter. However, the sector method has the disadvantage of being semi-automatic. The number of sectors to be considered must be determined by the experimenter. Too many sectors will tend to produce a fragmented picture of the occupied region, and too few sectors will cause that some fibers will fall outside of their region.

The relevance of Wang and Kernell (2001) to our work is in that, in essence, is the closest to the type of analysis we performed. More precisely, it tries to quantify the spatial localization of fibers and the region of the muscle cross-section they occupy. Like in the case of the sector method, our method is semi-automatic (the size of the counting grid must be determined by the experimenter). However, our method does provide a distribution function per se, which accounts for actual spatial location and number of fibers. The vector method provides a general direction in which fibers are located, but does not provide a sense of how fibers are distributed around the head of the mass vector. Our method specifically tells the experimenter where fibers are located within the muscle cross-section.

### 8.2. Dissimilarity Quantifiers

It is evident from our results that distance measurements show a tendency to increase as the number of points in the data set increases. In contrast, angle measurements seem to stabilize themselves as the number of data points increases, that is as more information is known about the data sets. Moreover, based on angle measurements alone we see that, as the data set increases (and thus becomes more defined), the pair ring vs. ball became the more similar pair among all, followed the ball vs. sum, and the ball vs. cross. Nonetheless, for these three pairs of shapes the angle between distributions is around or above 60 degrees which is two thirds of 90 degrees (maximum transversality) and four thirds of 45 degrees (halfway between colinearity and perpendicularity), therefore we think this is still clear evidence that the angle quantifier can serve as a tool to tell apart between two images. Note also that from the start the angle quantifier was able to distinguish the ring from the cross, the ring from the sum, and the cross from the sum, returning angles closer to 90 degrees.

The above discussion suggests that the angle quantifier is helpful in telling apart data sets which are uniformly distributed in all directions and highly localized (the ring), from data sets which are uniformly distributed and highly localized in specific directions (the sum and the cross), and even two data sets in the latter category so far as they are distributed along two distinct directions.

Currently, our distance quantifier depends strongly on the data size, but this can be fixed in the manner we suggest in the next paragraph. Right now, we want to make our point that angle and distance quantifiers are complementary dissimilarity quantifiers, in other words, they must both be used by the researcher who is trying to set apart two data sets based on the geometry of their spatial distributions. The argument is rather evident: based on Tables 1, 2, we see that the angle quantifier did not perform so as well setting aside the ring from the ball, the ball from the cross, and the ball from the sum, as it did setting apart the ring from the cross, the ring from the sum, and the cross from the sum. For instance, the angle value in the ring vs ball with 4,000 points is just above sixty degrees, whereas for a data set of the same size, the angle value in the ring vs sum is about 81 degrees, this amounts to a roughly 20 degree difference, and we perhaps “lose confidence” in the angle quantifier. But when we look at those same cases, the distance measurement for the ring vs. ball pair is 273, whereas for the ring vs. sum pair is 210, which tells us that the distance quantifier is better at distinguishing one data set from the other in the ring vs. ball pair, by a difference of over 60 units, so we “gain confidence” in this quantifier. In essence, when we think one quantifier is not performing to our standards, the other may be doing a better job.

To fix the scaling effect of the data set on the distance (larger data sets seem to yield larger values of the distance), we propose to modify our definition of the distance quantifier in the following way which completely eliminates that effect. Let

$$\begin{array}{l} D=d/\left(1+d\right), \end{array}$$

where *d* is the distance as calculated by (Equation S19, Supplementary Material). *D* is bounded below by zero and above by one and it is well-known in functional analysis that it also satisfies the properties of a metric. We are currently testing this quantifier and our preliminary results suggest that it is more subtle to appreciate differences in distance readings with this quantifier.

These preliminary results on artificial data suggest that the method of construction of distribution functions, as well as their “measure of dissimilarity” (distance and angle), are adequate tools to distinguish among trends of spread and clustering within the data. The case presented here is that of two-dimensional data, but of course the methodology lends itself to study the case of higher-dimensional data, with straightforward modifications on the sample covariance matrix.

Lastly, we must note that, whereas the sample covariance matrix suffers from the effect of “high dimensionality of the data” (it is a square matrix of size *d*, where *d* is the dimension of the data vectors), distance and angle evaluations solely depend on kernel evaluations which are convenient from a computational standpoint.

### 8.3. Fiber Type Organization and Distribution on the EDLm Fascicles

It is well known that skeletal muscles actively participate in the extension and flexion of articular joints (Lindstedt, 2016) as well as during changes in position (muscle length) or during generation of force (muscle strength) (Schappacher-Tilp et al., 2015). Such muscle properties allow vertebrate organisms to perform changes in posture and locomotion (Frontera and Ochala, 2015). It has been considered that each muscle is constituted by a variable proportion of slow, intermediate, and fast fibers. In the rat, the EDL muscle, an extensor muscle involved in the extension of the toes (2nd to 5th) and dorsiflexion of the ankle, is mostly conformed by fast-twitch fibers, meanwhile the soleus muscle, one of the flexor muscles of the calf, mainly contains slow-fiber twitch fibers (Armstrong and Phelps, 1984; Soukup et al., 2002). In addition, it has been proposed that contractile properties of each individual muscle are closely related to the relative proportion of fibers types and to their intra-muscle distribution in the cross-sectional area, mainly in the medial part of the muscle (Myatt et al., 2011).

In this study, we analyzed the fractal organization of fiber types in fascicles of the EDLm from well-nourished and undernourished rats. Our previous results (Vázquez-Mendoza et al., 2017) indicate that each fascicle in the EDLm, from both well-nourished and undernourished rats, showed a particular composition of fibers types. The relative proportion of intermediate and fast fibers in undernourished fascicles F2 and F4 had no significant differences with that of control fascicles, while the relative proportion of fiber types in fascicles F3 and F5 showed notorious differences with respect to controls (see Table 1), being the sequential order of fascicles affected by chronic undernutrition as follows: F3>F5>F4=F2. In the present study, we found that the calculated values of *a* index, *D* and *c* parameters corresponding to intermediate and fast fiber types were practically similar between control and undernourished fascicles (F2, F3, F4, and F5), indicating that fibers are organized in clusters over the transverse area of each fascicle. Meanwhile, only fast fibers in the undernourished F3 showed significant differences in fractal parameter *D*, as compared to those of control muscles, suggesting that fast fibers in the undernourished F3 are slightly less organized in clusters than in control ones. Because of the latter, it could be proposed that such fiber type cluster-organization is used as a mechanism to increase muscle efficiency (Myatt et al., 2011). Then, this change in the organization of fast fiber in the F3 could imply a change in the efficiency of the EDLm.

Although fiber organization was similar between conditions, fiber type distribution analysis showed that chronic undernutrition modifies the intra-fascicular fiber type distribution in the fascicles F3, F4, and F5. As fiber types distributions within a muscle are crucial to its functioning (Burkholder et al., 1994), these changes could induce alterations in muscle functioning. Thus, chronic undernutrition could be changing the efficiency and functioning of the EDLm fascicles.

Altogether, our observations indicate that chronic undernutrition exerts a more complex effect that just on the fiber type composition, finding a differential effect among the EDLm fascicles. Also, there is a differential effect on the distribution of intermediate and fast fibers in the EDLm fascicles and only the fractal dimension or structure of fast fibers in F3 seems to be modified by chronic undernutrition. All these differential effects on the properties of EDLm fascicles could be related to their anatomical position within the muscle and the fiber type composition. The F2 is located in the anterior part, followed by the F3, then the F4, and finally, the F5 is in the posterior part (Balice-Gordon and Thompson, 1988). In our previous work (Vázquez-Mendoza et al., 2017), the F3 was the most affected by chronic undernutrition, in the fiber metabolism as well as in the fiber type, together with the F5 (in fiber type changes) in comparison with the F2 and F4. This probably is related to the similarities of fiber type percentages composition (Vázquez-Mendoza et al., 2017). However, our data do not allow us to explain why a chronic food deprivation evoked such differential action on the EDLm fascicles, particularly on fascicles F3 and F5. In addition, it remains to be elucidated how the alterations provoked by undernutrition on the composition and fractal organization of fiber types in F3 affect the extension of the third toe and dorsiflexion of the ankle during a particular motor act (e.g., during gait locomotion).

## 9. Conclusion and Future Applications

RBF distribution functions constitute not only a visual aid to, for example, assess muscle structure and organization in the form of fiber distribution, but they also provide quantitative means by which to distinguish spatial distribution of fiber types. Those means are our dissimilarity quantifiers, distance and angle, defined between pairs of distribution functions. The mathematics of these quantifiers rests soundly on learning theory, and ultimately on functional analysis. Our results on artificial data suggest that distance and angle are dissimilarity quantifiers that complement one another. The angle quantifier is able to set apart data sets that spread along definite linearly independent directions in space or sets that spread along definite directions from sets that are uniformly spread along all directions while forming a single coherent cluster pattern (e.g., a ring). However, the angle quantifier is less able to set apart sets that are uniformly spread say, in the ring or in the ball type-of patterns, it is in these circumstances when the distance quantifier may be a better tool to distinguish between the two sets.

Other scenarios where the proposed method can be used is in current research oriented to reveal possible structural alterations of muscles provoked by traumatic processes, such as spinal cord injury, motor nerve damage, multiparity, or undernutrition/obesity. Of course, one may also use other histochemical techniques.

Finally, we would like to mention that although the problem that motivated this work comes from physiology, we hope that, given the potential to use the distance and angle quantifiers with high-dimensional data, the mathematical tools herein developed can also serve in other fields of Computational Biology.

## Data Availability Statement

All datasets generated for this study are included in the article/Supplementary Material.

## Ethics Statement

The animal study was reviewed and approved by All experiments were performed in accordance with the Guide for the Care and Use of Laboratory Animals (National Research Council, 2010; National Institutes of Health, Bethesda, MD, USA; Animal Welfare Assurance #A5036-01). The animal protocols were approved by the Institutional Bioethical Committee for the Care and Handling of Laboratory Animals (UPEAL-Protocol 013–02, CINVESTAV).

## Author Contributions

ER-T, JV-R, KL-G, SQ-G, and IJ-E conceived and designed the study. JV-R developed the theory for the distribution functions and dissimilarity quantifiers. EV-M carried out the experiments and analyzed the biological data. GC-F created the Matlab code for generating the synthetic data, binary images, distribution functions, and dissimilarity quantifiers. All authors contributed in writing the manuscript.

## Conflict of Interest

The authors declare that the research was conducted in the absence of any commercial or financial relationships that could be construed as a potential conflict of interest.
