# Supplementary material for: Chronic Undernutrition Differentially Changes Muscle Fiber Types Organization and Distribution in the EDL Muscle Fascicles
Source: Front Physiol. 2020 Jul 23;11:777. doi: 10.3389/fphys.2020.00777 (PMC7396705; doi:10.3389/fphys.2020.00777)
Supplement: Supplementary file 2 [file Data_Sheet_1.pdf]

Supplementary Material: Chronic Undernutrition Differentially Changes Muscle Fiber Types Organization and Distribution in the EDL Muscle Fascicles

1 SUPPLEMENTARY DATA

1.1 On the method of construction

We have structured this subsection in a series of observations with the intention to gradually gain insight into the distribution function defined in (7).

1. If a matrix Σ is positive definite (i.e., symmetric, such that $\xi^t\Sigma\xi \geq 0$ and equality holding if and only if ξ is the zero vector), then its inverse Σ^{-1} exists and is positive definite as well. The covariance matrices Σ_{ij} used in this work are always positive definite (cf. (5) and points (ii) and (iii) in the previous section), this guarantees that all functions ϕ_{ij} have bell-shaped graphs.

2. Lemma 1: if Σ is a positive definite matrix, the quadratic form $d : \mathbb{R}^2 \times \mathbb{R}^2 \to \mathbb{R}$, defined below is a metric in $\mathbb{R}^2$.

$$d^2(\bar{\mathsf{z}}, \mathsf{z}) = (\mathsf{z} - \bar{\mathsf{z}})^t\Sigma^{-1}(\mathsf{z} - \bar{\mathsf{z}}) \tag{S1}$$

Proof: clearly, $d(\bar{\mathsf{z}}, \mathsf{z}) = d(\mathsf{z}, \bar{\mathsf{z}})$ and since Σ^{-1} is positive definite $d^2(\bar{\mathsf{z}}, \mathsf{z}) \geq 0$ for any $\bar{\mathsf{z}}$ and z in $\mathbb{R}^2$. Also, as a positive definite matrix, Σ^{-1} is unitarily diagonalizable and its eigenvalues are positive. Therefore there exists an invertible matrix U whose inverse is its transpose matrix, such that $U^t\Sigma U = \text{diag}(\mu_1, \mu_2)$, with $\mu_1 > 0$ and $\mu_2 > 0$ (in fact, $\mu_i = \lambda_i^{-1}$, where λ_i are the eigenvalues of Σ). Thus if $d^2(\bar{\mathsf{z}}, \mathsf{z}) = 0$ and $(\mathsf{z} - \bar{\mathsf{z}}) = U\xi$, one has $d^2 = (\mathsf{z} - \bar{\mathsf{z}})^t\Sigma^{-1}(\mathsf{z} - \bar{\mathsf{z}}) = \xi^t\text{diag}(\mu_1, \mu_2)\xi = \mu_1\xi_1^2 + \mu_2\xi_2^2$ ($\mu_i > 0$, $i = 1, 2$). Thus $d^2 = 0$ if and only if $\xi_1 = \xi_2 = 0$, that is $\xi = 0$, thus $U(\mathsf{z} - \bar{\mathsf{z}}) = 0$ and since U is invertible one has $\mathsf{z} - \bar{\mathsf{z}} = 0$. Thus $d^2 = 0$ if and only if $\mathsf{z} = \bar{\mathsf{z}}$. To finish showing that d is a metric we need to establish the triangle inequality. First notice that for any η in $\mathbb{R}^2$ one has that $\eta^t\Sigma^{-1}\eta = \eta^tU\text{diag}(\mu_1, \mu_2)U^t\eta = \eta^tU\text{diag}(\mu_1^{1/2}, \mu_2^{1/2})\text{diag}(\mu_1^{1/2}, \mu_2^{1/2})U^t\eta = \|\text{diag}(\mu_1^{1/2}, \mu_2^{1/2})U^t\eta\|^2$. Now, let z, $\bar{\mathsf{z}}$ and z' in $\mathbb{R}^2$, thus

$$\begin{aligned}
d(\bar{\mathsf{z}}, \mathsf{z}) &= \|\text{diag}(\mu_1^{1/2}, \mu_2^{1/2})U^t(\mathsf{z} - \bar{\mathsf{z}})\| \\
&= \|\text{diag}(\mu_1^{1/2}, \mu_2^{1/2})U^t(\mathsf{z} - \mathsf{z}' + \mathsf{z}' - \bar{\mathsf{z}})\| \\
&= \|\text{diag}(\mu_1^{1/2}, \mu_2^{1/2})U^t(\mathsf{z} - \mathsf{z}') + \text{diag}(\mu_1^{1/2}, \mu_2^{1/2})U^t(\mathsf{z}' - \bar{\mathsf{z}})\| \\
&\leq \|\text{diag}(\mu_1^{1/2}, \mu_2^{1/2})U^t(\mathsf{z} - \mathsf{z}')\| + \|\text{diag}(\mu_1^{1/2}, \mu_2^{1/2})U^t(\mathsf{z}' - \bar{\mathsf{z}})\| \\
&= d(\mathsf{z}', \mathsf{z}) + d(\bar{\mathsf{z}}, \mathsf{z}')\,;
\end{aligned}$$

the inequality follows from the triangle inequality of the euclidean norm. QED.

3. Lemma 2.: if d is a metric, then

$$\kappa(\bar{\mathsf{z}},\mathsf{z}) = \exp(-2^{-1}d^2(\bar{\mathsf{z}},\mathsf{z}))\,, \tag{S2}$$

is a *kernel function* (cf. Cristianini et al. (2000), ch.3). When d is the euclidean distance κ is basically a *Gaussian radial basis function* (Schölkopf et al. (2004) p.41), that is

(i) κ is symmetric: $\kappa(\bar{\mathsf{z}},\mathsf{z}) = \kappa(\mathsf{z},\bar{\mathsf{z}})$, and

(ii) for any collection of vectors $\{\mathsf{u}_1,\ldots,\mathsf{u}_N\}$ in $\mathbb{R}^2$, $\mathsf{b}^t\mathbb{K}\mathsf{b} \geq 0$ for all b in $\mathbb{R}^N$, where $\mathbb{K}$ is a matrix with components $\mathbb{K}_{ij} = \kappa(\mathsf{u}_i,\mathsf{u}_j)$.

Proof: symmetry of κ is a consequence of the symmetry of the metric d. To prove (ii) first note that $0 < \kappa(\bar{\mathsf{z}},\mathsf{z}) \leq 1$, and that $\kappa(\bar{\mathsf{z}},\mathsf{z}) = 1$ if and only if $\bar{\mathsf{z}} = \mathsf{z}$. Then if $\mathsf{b} = (b_1,\ldots,b_N)$ and $\mathbb{K}$ is as defined in (ii), we have

$$\begin{aligned}\mathsf{b}^t\mathbb{K}\mathsf{b} &= \sum_{i=1}^N b_i^2 + 2\sum_{i<j} b_ib_j\kappa(\mathsf{u}_i,\mathsf{u}_j) \geq \sum_{i=1}^N b_i^2 - 2\sum_{i<j}|b_i|\,|b_j|\kappa(\mathsf{u}_i,\mathsf{u}_j)\\ &\geq \sum_{i=1}^N b_i^2 - 2\sum_{i<j}|b_i|\,|b_j| = \sum_{i<j}(|b_i|-|b_j|)^2 \geq 0\,.\end{aligned} \tag{S3}$$

Suppose that there is a vector b such that $\mathsf{b}^t\mathbb{K}\mathsf{b} = 0$, then the series of inequalities in (S3) are all equalities. In particular the last inequality implies that $|b_i| = |b_j|$ for all $i \neq j$, say $|b_i| = a^2$ for all i. Then the second to last inequality implies that

$$\sum_{i<j} a^4(1-\kappa(\mathsf{u}_i,\mathsf{u}_j)) = 0\,, \tag{S4}$$

for *any* collection of vectors $\mathsf{u}_i's$. Note that every summand in (S4) is non-negative, therefore to add up to zero necessarily each one must be zero. If the collection of $\mathsf{u}_i's$ consists of distinct vectors, $\kappa(\mathsf{u}_i,\mathsf{u}_j) < 1$, thus $a = 0$, so $|b_i| = 0$, that is $b_i = 0$ for all i and thus b is the zero vector (in which case $\mathsf{b}^t\mathbb{K}\mathsf{b}$ is a nondegenerate quadratic form). QED.

4: Define $\kappa_{ij} : \mathbb{R}^2 \times \mathbb{R}^2 \to \mathbb{R}$ as follows,

$$\kappa_{ij}(\mathsf{y},\mathsf{z}) = \exp\Big(-2^{-1}(\mathsf{z}-\mathsf{y})^t\Sigma_{ij}^{-1}(\mathsf{z}-\mathsf{y})\Big)\,, \tag{S5}$$

where Σ_{ij} is as before the sample covariance matrix for cell C_{ij} and z and y are any vectors in $\mathbb{R}^2$. Define the real linear function space

$$\mathcal{L}_{ij} = \text{span}\{\kappa_{ij}(\mathsf{y},\cdot) : \mathsf{y} \in \mathbb{R}^2\}\,. \tag{S6}$$

Lemma 3: $\mathcal{L}_{ij}$ is an inner-product space. For f and g in $\mathcal{L}_{ij}$ given by

$$f(\mathsf{z}) = \sum_{k=1}^m D_k\kappa_{ij}(\mathsf{u}_k,\mathsf{z}) \quad\text{and}\quad g(\mathsf{z}) = \sum_{\ell=1}^n E_\ell\kappa_{ij}(\mathsf{v}_\ell,\mathsf{z}), \tag{S7}$$

their inner product $(f,g)_{ij}$ is defined by

$$(f,g)_{ij} = \sum_{k=1}^{m}\sum_{\ell=1}^{n} D_k E_\ell \kappa_{ij}(\mathsf{u}_k, \mathsf{v}_\ell)\,. \tag{S8}$$

Proof: linearity verifies straightforwardly, whereas symmetry and non-degeneracy follow immediately from the previous lemma. QED.

5. Being an inner product space $\mathcal{L}_{ij}$ is a normed and thus also a metric space: for each f in $\mathcal{L}_{ij}$ as above, we can define its norm $\|f\|_{ij}$ as follows,

$$\|f\|_{ij}^2 = (f,f)_{ij} = \sum_{k=1}^{m}\sum_{\ell=1}^{m} D_k D_\ell \kappa_{ij}(\mathsf{u}_k, \mathsf{u}_\ell)\,. \tag{S9}$$

If g in $\mathcal{L}_{ij}$ is as above then we can define the distance between f and g, $d_{ij}(f,g)$, as

$$d_{ij}^2(f,g) = (f-g, f-g) = \|f\|_{ij}^2 + \|g\|_{ij}^2 - 2(f,g)_{ij}\,. \tag{S10}$$

Note in particular that $\phi_{ij}(\mathsf{z}) = \kappa_{ij}(\bar{\mathsf{z}}_{ij}, \mathsf{z})$ and thus it is an element of $\mathcal{L}_{ij}$, moreover

$$\|\phi_{ij}\|_{ij}^2 = \kappa_{ij}(\bar{\mathsf{z}}_{ij}, \bar{\mathsf{z}}_{ij}) = 1\,. \tag{S11}$$

6. By Cauchy-Schwarz inequality, the cosine of the angle θ between f and g is defined by

$$\cos\theta = \frac{(f,g)_{ij}}{\|f\|_{ij}\|g\|_{ij}}\,. \tag{S12}$$

f and g are said to be perpendicular if $\cos\theta = 0$.

7. Let $\mathcal{L}_N = \bigoplus\{\mathcal{L}_{ij} : i,j = 1,\ldots,N\}$ represent the real space whose elements are linear combinations of functions drawn from the spaces $\mathcal{L}_{ij}$. We show that $\mathcal{L}_N$ is an inner product space. Let elements in $\mathcal{L}_N$

$$F(\mathsf{z}) = \sum_{i=1}^{N}\sum_{j=1}^{N}\sum_{k=1}^{m_{ij}} D_k^{ij}\kappa_{ij}(\mathsf{u}_k^{ij}, \mathsf{z}) \quad \text{and} \quad G(\mathsf{z}) = \sum_{i=1}^{N}\sum_{j=1}^{N}\sum_{\ell=1}^{n_{ij}} E_\ell^{ij}\kappa_{ij}(\mathsf{v}_\ell^{ij}, \mathsf{z})\,, \tag{S13}$$

where $\{\mathsf{u}_k^{ij} : k = 1,\ldots,m_{ij};\, i,j = 1,\ldots,N\}$ and $\{\mathsf{v}_\ell^{ij} : \ell = 1,\ldots,n_{ij};\, i,j = 1,\ldots,N\}$ are arbitrary collections of vectors in $\mathbb{R}^2$. The inner product between F and G is defined by

$$(F,G)_N = \sum_{i=1}^{N}\sum_{j=1}^{N}\Big(\sum_{k=1}^{m_{ij}} D_k^{ij}\kappa_{ij}(\mathsf{u}_k^{ij}, \mathsf{z}), \sum_{\ell=1}^{n_{ij}} E_\ell^{ij}\kappa_{ij}(\mathsf{v}_\ell^{ij}, \mathsf{z})\Big)_{ij} = \sum_{i=1}^{N}\sum_{j=1}^{N}\sum_{k=1}^{m_{ij}}\sum_{\ell=1}^{n_{ij}} E_\ell^{ij} D_k^{ij}\kappa_{ij}(\mathsf{u}_k^{ij}, \mathsf{v}_\ell^{ij})\,. \tag{S14}$$

Symmetry, linearity, and non-degeneracy of this inner-product are a straight-forward consequence of the fact that these properties were already established for the individual inner-products $(\cdot,\cdot)_{ij}$. Also note that, by this construction, the factor spaces $\mathcal{L}_{ij}$ that make up $\mathcal{L}_N$ are mutually orthogonal subspaces of $\mathcal{L}_N$.

8. Similarly, as for $\mathcal{L}_{ij}$, being an inner product space, $\mathcal{L}_N$ is a normed space and also a metric space. Indeed, if F and G are defined as in (S13) then

$$\|F\|_N^2 = (F,F)_N = \sum_{i=1}^{N}\sum_{j=1}^{N}\Big\|\sum_{k=1}^{m_{ij}} D_k^{ij}\kappa_{ij}(\mathsf{u}_k^{ij},\mathsf{z})\Big\|_{ij}^2 = \sum_{i=1}^{N}\sum_{j=1}^{N}\sum_{k=1}^{m_{ij}}\sum_{\ell=1}^{m_{ij}} D_k^{ij}D_\ell^{ij}\kappa_{ij}(\mathsf{u}_k^{ij},\mathsf{u}_\ell^{ij})\,, \tag{S15}$$

and

$$\begin{aligned} d_N^2(F,G) = (F-G,F-G)_N &= \sum_{i=1}^{N}\sum_{j=1}^{N} d_{ij}^2\Big(\sum_{k=1}^{m_{ij}} D_k^{ij}\kappa_{ij}(\mathsf{u}_k^{ij},\mathsf{z}),\sum_{\ell=1}^{n_{ij}} E_k^{ij}\kappa_{ij}(\mathsf{v}_\ell^{ij},\mathsf{z})\Big) \\ &= \|F\|_N^2 + \|G\|_N^2 - 2(F,G)_N\,. \end{aligned} \tag{S16}$$

Likewise, similarly as in (S12) the cosine of the angle θ between F and G is defined as

$$\cos\theta = \frac{(F,G)_N}{\|F\|_N\|G\|_N}\,. \tag{S17}$$

9. The distribution function Φ defined in (7) belongs to $\mathcal{L}_N$. Φ only takes one element $\mathsf{u}^{ij} = \bar{\mathsf{z}}_{ij}$ per cell C_{ij} (i.e. per each pair (i,j)), and given that the functions ϕ_{ij} are pairwise orthogonal and unitary (cf. (S11)), one has (recall that $R_{ij} = |S_{ij}|$)

$$\|\Phi\|_N^2 = \sum_{i=1}^{N}\sum_{j=1}^{N} R_{ij}^2\,. \tag{S18}$$

In fact, for purposes of the next section, let $\Psi(\mathsf{z}) = \sum_{i=1}^{N}\sum_{j=1}^{N} T_{ij}\psi_{ij}(\mathsf{z})$, where the coefficients T_{ij} are real numbers. $\psi_{ij}(\mathsf{z}) = \kappa_{ij}(\bar{\mathsf{z}}'_{ij},\mathsf{z})$ and the $\bar{\mathsf{z}}'_{ij}$ are vectors in $\mathbb{R}^2$. Ψ is thus a function like Φ (only takes one vector u_{ij} per each pair (i,j)). In this particular case

$$d^2(\Phi,\Psi) = \|\Phi\|_N^2 + \|\Psi\|_N^2 - 2(\Phi,\Psi)_N = \sum_{i=1}^{N}\sum_{j=1}^{N}(R_{ij}^2 + T_{ij}^2 - 2R_{ij}T_{ij}\kappa_{ij}(\bar{\mathsf{z}}_{ij},\bar{\mathsf{z}}'_{ij}))\,, \tag{S19}$$

and (S17) becomes

$$\cos\theta = \frac{\sum_{i=1}^{N}\sum_{j=1}^{N} R_{ij}T_{ij}\kappa_{ij}(\bar{\mathsf{z}}_{ij},\bar{\mathsf{z}}'_{ij})}{\Big(\sum_{i=1}^{N}\sum_{j=1}^{N} R_{ij}^2\Big)^{1/2}\Big(\sum_{i=1}^{N}\sum_{j=1}^{N} T_{ij}^2\Big)^{1/2}} \tag{S20}$$

10. Being an inner-product space $\mathcal{L}_N$ can be completed, that is there exists a Hilbert space $\mathcal{H}_N$ and an isomorphism from $\mathcal{L}_N$ to a dense subspace of $\mathcal{H}_N$ (the latter being unique except for isomorphisms, cf. Kreyszig (1978), p.139). This observation endows $\mathcal{L}_N$ with a rich setting in which, for instance, it is possible to consider sequences of functions and speak of their convergence. It also opens the possibility of taking the limit $N \to \infty$. The latter scenario may be relevant when working with data sets which are not discrete, such as sets of functions defined on subdomains of the plane. If explicit expressions for those functions are not available, or they are available but are of little to no practical use, one can try to approximate such functions by elements in $\mathcal{L}_N$ and then study in which sense to take the limit $N \to \infty$.

11. Finally, we bring attention to the fact that our functions ϕ_{ij} are not compactly supported and this causes them to "leak" to other cells of the grid. More precisely, the idea behind thinking of Φ as a smoothed

histogram is that Φ will assume the value $|S_{ij}|$ at $\bar{\mathsf{z}}_{ij}$ (centroid of S_{ij}). The latter will not be the case since the functions ϕ_{ij} that make up Φ do not have compact support. In the case study of this manuscript, this is not of significant concern since the Gaussian functions ϕ_{ij} decay rapidly enough to make their contribution to Φ negligible outside of their respective cells. Nonetheless, this point can be corrected if one is willing to pay a certain extra computational cost. Indeed, to make Φ assume the exact value $|S_{ij}|$ when $z = \bar{\mathsf{z}}_{ij}$ ($\Phi(\mathsf{z}_{ij}) = |S_{ij}|$), the coefficients R_{ij} must solve the following $N^2 \times N^2$ linear system:

$$|S_{ij}| = \sum_{k=1}^{N}\sum_{\ell=1}^{N} R_{k\ell}\kappa_{k\ell}(\bar{\mathsf{z}}_{k\ell}, \bar{\mathsf{z}}_{ij})\,, \quad i = 1, \ldots, N\,, \quad j = 1, \ldots, N\,. \tag{S21}$$

Note that $|S_{ij}| = \mathrm{tr}(RK^t(\mathsf{z}_{ij}))$, where R and $K(\mathsf{z}_{ij})$ are square matrices of size N whose (k, ℓ) components are $R_{k\ell}$ and $\kappa_{k\ell}(\bar{\mathsf{z}}_{k\ell}, \bar{\mathsf{z}}_{ij})$, respectively.

REFERENCES

Cristianini, N., Shawe-Taylor, J., et al. (2000). *An introduction to support vector machines and other kernel-based learning methods*. Cambridge University Press, Cambridge, UK.

Kreyszig, E. (1978). *Introductory functional analysis with applications*, volume 1. wiley New York.

Schölkopf, B., Tsuda, K., Vert, J.-P., Istrail, D. S., Pevzner, P. A., Waterman, M. S., et al. (2004). *Kernel methods in computational biology*. MIT press, Cambridge, MA.
